# Principles Governing Locomotion in Vertebrates: Lessons From Zebrafish

**DOI:** 10.3389/fncir.2018.00073

**Published:** 2018-09-13

**Authors:** Eva M. Berg, E. Rebecka Björnfors, Irene Pallucchi, Laurence D. Picton, Abdeljabbar El Manira

**Affiliations:** Department of Neuroscience, Karolinska Institute (KI), Stockholm, Sweden

**Keywords:** spinal cord, excitatory interneurons, neural networks, plasticity, motor behavior and motor control

## Abstract

Locomotor behaviors are critical for survival and enable animals to navigate their environment, find food and evade predators. The circuits in the brain and spinal cord that initiate and maintain such different modes of locomotion in vertebrates have been studied in numerous species for over a century. In recent decades, the zebrafish has emerged as one of the main model systems for the study of locomotion, owing to its experimental amenability, and work in zebrafish has revealed numerous new insights into locomotor circuit function. Here, we review the literature that has led to our current understanding of the neural circuits controlling swimming and escape in zebrafish. We highlight recent studies that have enriched our comprehension of key topics, such as the interactions between premotor excitatory interneurons (INs) and motoneurons (MNs), supraspinal and spinal circuits that coordinate escape maneuvers, and developmental changes in overall circuit composition. We also discuss roles for neuromodulators and sensory inputs in modifying the relative strengths of constituent circuit components to provide flexibility in zebrafish behavior, allowing the animal to accommodate changes in the environment. We aim to provide a coherent framework for understanding the circuitry in the brain and spinal cord of zebrafish that allows the animal to flexibly transition between different speeds, and modes, of locomotion.

## Introduction

Locomotion is one of the most essential behaviors in an animal’s repertoire, enabling, among other things: foraging, feeding, escaping a threat and procreating. Locomotor behaviors such as swimming, walking, crawling and flying are all generated by networks of neurons known as central pattern generators (CPGs), located in the spinal cord in vertebrates and in the ventral nerve cord in invertebrates (Brown, 1911; Wilson, 1961; Grillner, 1981). These networks have been studied extensively in a wide range of species across the phylogenetic spectrum (Getting et al., 1980; Robertson and Pearson, 1985; Roberts et al., 1998; Eisenhart et al., 2000; Grillner, 2003; Gjorgjieva et al., 2013; Kiehn, 2016).

Studies in each species come with their own set of advantages and disadvantages. The relative simplicity and accessibility of the nervous system of non-mammalian vertebrates, like the zebrafish, allows for an in-depth analysis of neuronal connectivity and cellular circuit details (Fetcho et al., 2008; Gebhardt et al., 2013; Grillner and El Manira, 2015; Koyama et al., 2016; Knafo and Wyart, 2018) that, at the moment, cannot be achieved in mammals. As one of few preparations, the zebrafish is accessible to intracellular electrophysiological recordings from larval to adult stages, allowing a detailed investigation and comparison of the neuronal organization of locomotion during the course of development (McLean et al., 2007, 2008; Gabriel et al., 2011; Ausborn et al., 2012; Wang and McLean, 2014). Furthermore, zebrafish larvae have the additional benefit of being completely transparent, allowing broad neuronal activity to be monitored even in intact and freely behaving animals (Ahrens et al., 2013; Cong et al., 2017; Dal Maschio et al., 2017; Kim et al., 2017; Haesemeyer et al., 2018). These unique benefits of the zebrafish model system have permitted numerous insights into the principles of the locomotor circuitry controlling swimming which could serve as a conceptual framework to other vertebrate model systems.

These insights provide us with an understanding of CPG development and function that often apply to the entire vertebrate lineage. The nervous system across vertebrates is remarkably similar in its basic structure (Jessell, 2000; Goulding, 2009; Tümpel et al., 2009; Tripodi and Arber, 2012; Hirashima and Adachi, 2015; D’Elia and Dasen, 2018) and shares a broad organization of brain areas because it needs to solve many of the same fundamental problems in our common world. Of course, many brain areas evolved considerably over time, but for an ancient and essential behavior like locomotion, many features appeared early in evolution and have been conserved over time despite variations (Katz, 2016). Studying a wide range of species therefore not only makes sense from a comparative point of view, but also because organization principles discovered in early evolving organisms are likely to be shared by late evolving species, albeit integrated into larger networks (Orlovsky et al., 1999; Hooper and Büschges, 2017).

The basic architecture of the locomotor CPG in aquatic vertebrates has been described in depth in lamprey (Grillner, 2003) and in *Xenopus* tadpoles (Roberts et al., 2010), which comprises motoneurons (MNs) that activate muscle; ipsilateral excitatory INs that provide excitatory drive; inhibitory commissural INs that ensure left-right alternation, and ipsilateral inhibitory INs that contribute to burst termination. A conceptual framework for the mammalian CPG has also been inferred and developed from this overall ground plan (Kiehn, 2011). Zebrafish share this overall CPG architecture, but in this review we will not include a broad conceptual CPG circuit diagram which has been reviewed elsewhere (e.g., Grillner, 2003; Büschges et al., 2011; Kiehn, 2016), but instead focus on the detailed connectivity and activity patterns that have been established electrophysiologically in combination with anatomical studies in zebrafish. This review will summarize the insights gained from studying zebrafish into the organization of the spinal locomotor circuits, their activation by descending projections and how their activity is refined by neuromodulation and sensory inputs.

## Neuronal Diversity and Transcriptional Code

**Figure 1:**
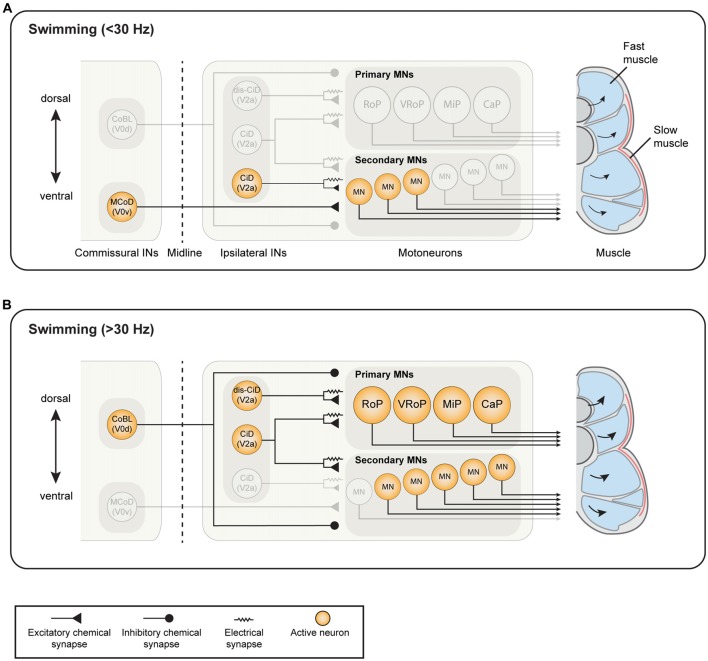
Known active neuron types and established connectivity within the spinal swim central pattern generator (CPG) network of larval zebrafish. **(A)** During slow swimming (<30 Hz), only the most ventral motoneurons (MNs) are active, which receive excitatory drive from ventrally-located circumferential descending (CiD) interneurons (INs; ventral V2a INs). Commissural excitatory INs multipolar commissural descendings (MCoDs), a subtype of V0v IN, also provide excitation to secondary motor neurons from the contralateral side. **(B)** During fast swimming (>30 Hz), the ventral MNs remain active but more dorsal secondary MNs and primary MNs also become recruited. Both the ventral CiD and MCoDs are actively de-recruited, whereas the dorsally-located CiDs and displaced dorsal CiDs (dis-CiD) become active. The displaced CiDs provide selective excitation to primary motor neurons. Note that several IN types known to be active during swimming are not displayed as their roles are less well defined.

In both larval and adult zebrafish, considerable effort has been invested into characterizing the various spinal neuron classes to determine their functional roles within the locomotor CPGs (see Table 1). The initial description of the different components of the CPG was based on anatomical analysis in larval zebrafish, and different neurons were classified based on their morphological characteristics such as soma shape, size and position, extent of the dendritic tree, axonal trajectory and time of axogenesis (Bernhardt et al., 1990; Hale et al., 2001). These approaches led to the identification of two major classes of MNs based on their time of development: the early born primary MNs consisting of 3–4 neurons per spinal hemi-segment (Myers, 1985; Myers et al., 1986; Eisen et al., 1989; Bernhardt et al., 1990; Eisen, 1999; Menelaou and McLean, 2012) and the late born secondary MNs (Figure 1; van Raamsdonk et al., 1983; Myers, 1985; Bernhardt et al., 1990). In addition, eight different classes of INs were described (Bernhardt et al., 1990; Hale et al., 2001; Drapeau et al., 2002). The neurotransmitter phenotypes of these IN classes were later determined in larval zebrafish using immunohistochemistry, extending the already available information and facilitating the analysis of their function. The glutamatergic neurons identified include the circumferential descending (CiD), the multipolar commissural descending (MCoD), the unipolar commissural descending (UCoD), the commissural primary ascending (CoPA) and some of the commissural secondary ascending (CoSA). The glycinergic neurons identified include the circumferential ascending (CiA), some CoSAs, the commissural longitudinal bifurcating (CoBL) and the commissural longitudinal ascending (CoLA). Two types of GABAergic neurons were also identified: the dorsal longitudinal ascending (DoLA) and the Kolmer-Agduhr (KA) neurons, with the latter corresponding to the cerebrospinal fluid-contacting neurons (CSF-CNs; Higashijima et al., 2004a,b,c; Djenoune et al., 2014).

**Table 1:** A comparison of the swim pattern, muscle properties and swim network organization of the larval and juvenile/adult zebrafish.

	Larval zebrafish (4–6 dpf)	Juvenile/Adult zebrafish (>7 weeks)
Swim pattern	• Burst and glide (Saint-Amant and Drapeau, 1998; Budick and O’Malley, 2000; Buss and Drapeau, 2001)	• Continuous episodes (Müller et al., 2000; Gabriel et al., 2011)
Swim frequencies	• 20–60 Hz (Mean: ~35 Hz; Saint-Amant and Drapeau, 1998; Buss and Drapeau, 2001; Masino and Fetcho, 2005)	• 1–21 Hz (Mean: ~6 Hz; Kyriakatos et al., 2011; Ampatzis et al., 2013)
Muscle fiber types	• Embryonic red (slow), white (fast; van Raamsdonk et al., 1982; Devoto et al., 1996; Jackson and Ingham, 2013)	• Red (slow), pink (intermediate), white (fast; van Raamsdonk et al., 1982; Devoto et al., 1996)
Motoneuron (MN) recruitment	Secondary MNs: (McLean et al., 2007) Sequential recruitment with speed Recruitment according to size principle Primary MNs: (Liu and Westerfield, 1988; McLean et al., 2007) Recruited at fast swim speeds and escape	Secondary MNs: (Gabriel et al., 2011; Ampatzis et al., 2013) Sequential recruitment with speed Recruitment not according to size principle Recruitment aligns to innervated muscle types Primary MNs: (Gabriel et al., 2011; Ampatzis et al., 2013) Recruited only during escape
Interneuron (IN) recruitment	V2a (CiD) and a subset of V0V (MCoD) that are recruited at slow speeds are actively inhibited at high speeds (McLean et al., 2007, 2008)	V2a and V0v recruited sequentially as speed increases and remain active (Ausborn et al., 2012; Ampatzis et al., 2014; Björnfors and El Manira, 2016)
Somatotopic organization	MNs: • Yes (Myers, 1985; Myers et al., 1986; Westerfield et al., 1986; McLean et al., 2007) INs: • Yes V0V (MCoD; McLean et al., 2007, 2008; McLean and Fetcho, 2009), V0d (CoBL; McLean et al., 2007), V1 (CiA; McLean et al., 2007), V2a (CiD; Kimura et al., 2006; McLean et al., 2007, 2008; McLean and Fetcho, 2009)	MNs: • Yes (Gabriel et al., 2011; Ampatzis et al., 2013) INs: • No: V0v (Björnfors and El Manira, 2016), V2a (Ausborn et al., 2012)
V2a (CiD) to MN connectivity	Electrical and chemical connections (Kimura et al., 2006) Displaced V2a selective for primary MNs (Svara et al., 2018)	Modular organization with mixed electrical and chemical synapses (Ampatzis et al., 2014; Song et al., 2016) Fast V2a selective for fast MNs Intermediate V2a selective for intermediate MNs Slow V2a selective for slow MNs

The anatomical- and neurotransmitter-based diversity of INs was subsequently extended using molecular markers for the different neuronal classes based on their developmental origins. In all vertebrates, the neuronal types develop according to a conserved plan orchestrated by the concentration gradient of morphogens from the floor and roof plates (Yamada et al., 1991; Ericson et al., 1995, 1997; Liem et al., 1997; Briscoe et al., 2000; Timmer et al., 2002; Chizhikov and Millen, 2004). There are five ventral progenitor domains, one giving rise to the MNs and four domains, termed V0-V3, giving rise to the CPG INs. In addition, there are six dorsal domains (dI1–dI6). These cardinal neuronal populations express specific transcription factors that has enabled the generation of specific transgenic animals to probe the function of whole spinal neuron populations related to each other through development (Goulding et al., 1993; Liem et al., 1995, 2000; Ericson et al., 1997; Briscoe et al., 2000; Jessell, 2000; Lee and Pfaff, 2001; Gross et al., 2002; Higashijima et al., 2004a; Lanuza et al., 2004; Kimura et al., 2006; Goulding, 2009; Satou et al., 2012; Gosgnach et al., 2017). It is becoming clear that the transcriptional code in the developing spinal cord is conserved across the vertebrate lineage.

Using the transcriptional code, some of the neuronal types described anatomically in larval zebrafish could be further characterized. Out of the ipsilaterally projecting INs, the glycinergic CiAs belong to the V1 population and express the transcription factor En1, while the CiDs correspond to the V2a population defined by the expression of the transcription factor Chx10 (Higashijima et al., 2004a; Kimura et al., 2006). The commissural INs belong to the V0 population that, among others, encompasses the glutamatergic V0v INs such as MCoDs and UCoDs and the inhibitory V0d INs such as CoBLs (Satou et al., 2012). The inhibitory V0d population seems morphologically homogeneous with all INs displaying bifurcating axonal projections regardless of whether they are glycinergic or GABAergic. By contrast, the excitatory V0v population is heterogeneous, with different morphological profiles that are linked to their temporal pattern of differentiation. The first differentiating V0v INs have ascending axons, followed by those with bifurcating axons and finally the descending ones such as MCoDs and UCoDs (Satou et al., 2012; Juárez-Morales et al., 2016).

## Developmental Changes in Swimming Pattern and Frequency Coupled to Muscle Development

The diversity of neurons can endow animals with a more flexible locomotor activity according to the behavioral needs at different developmental stages. Swimming allows zebrafish to move through their environment. It consists of alternating the activity of axial musculature on the right and left sides of the body, generating propulsive undulatory movements that propel the fish through the water (Jayne and Lauder, 1994; Budick and O’Malley, 2000; Müller et al., 2000). The ability of zebrafish to move at, and transition smoothly between, a relatively large span of swim frequencies has been studied throughout development using numerous techniques. Electrophysiological recordings from nerve bundles in the inter myotomal clefts on both sides of the body have shown that the activity is left-right alternating and, in addition, rostro-caudally propagating with a constant delay between segments. This pattern of activity is found at both larval stages (4–6 dpf; Saint-Amant and Drapeau, 1998; Buss and Drapeau, 2001; Masino and Fetcho, 2005), juvenile stages (4–6 weeks) and adult stages (7–15 weeks; Gabriel et al., 2008; Kyriakatos et al., 2011). However, swimming frequencies are higher in the larva, around 20–60 Hz with a mean frequency of 35.6 Hz, (Saint-Amant and Drapeau, 1998; Buss and Drapeau, 2001; Masino and Fetcho, 2005) and decrease drastically during development to 1–21 Hz with a mean of 6 Hz in the adult (Kyriakatos et al., 2011; Ampatzis et al., 2013).

**Figure 2:**
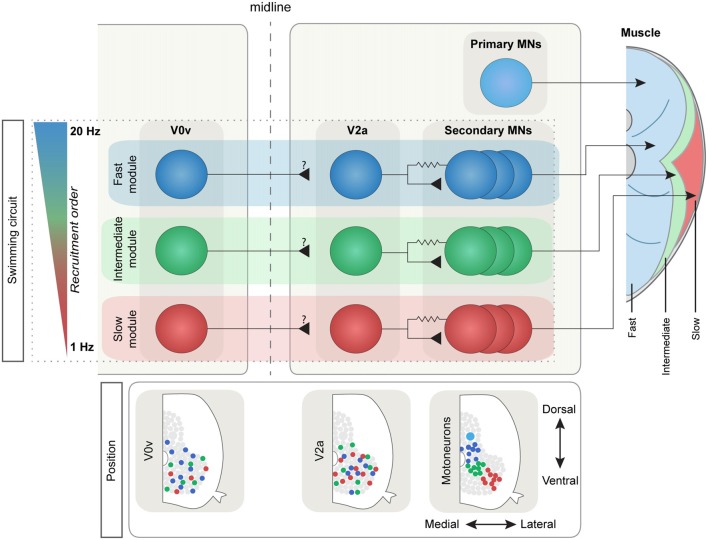
The modular swim network in adult zebrafish. The IN and MN populations in the adult zebrafish are subdivided into three main types depending on the recruitment frequency: slow (red), intermediate (green) and fast (blue). This subdivision reflects the three distinct muscle types that in zebrafish are spatially segregated (right): the anaerobic “white” fibers (in blue), the aerobic “red” fibers (in red) and the intermediate fibers (in green). The main ipsilateral excitatory drive comes from the V2a population that is connected preferentially to MNs belonging to the same module through mixed chemical and electrical synapses. The V0v population in adult zebrafish also shows a differential speed-depended recruitment order, although its connectivity has not been yet defined. Bottom: MN pools are spatially defined, while V0v and V2a INs have lost their topographical organization during development.

The observed decrease in overall swimming frequency during the development of zebrafish occurs in accordance with a change in the muscle fiber composition (compare Figure 1 for larval, Figure 2 for adult zebrafish, and Table 1). When fully developed, the adult zebrafish musculature consists of three distinct muscle fiber types: red, white and intermediate pink. Unlike in mammals, these muscle fiber types are distinctly segregated from each other, making them easier to study (Devoto et al., 1996; Jackson and Ingham, 2013). The red fiber types are aerobic and enriched in myoglobin, whereas the white are anaerobic, large diameter fibers. These distinct properties ensure specialization for low force, sustained movements or strong short duration movements, respectively. In between the red and the white fibers are the pink (or intermediate) fibers which have distinct immunohistochemical properties and likely originate from the fast-white fibers (van Raamsdonk et al., 1982). Larval zebrafish muscle is well characterized in terms of the gene regulatory mechanisms involved during development (Jackson and Ingham, 2013). Larval muscle is composed of two fiber types, so called thin flat superficial fibers, which are red fibers, and large, polygonal deep fibers, which are white fibers. Although the superficial red fibers in the larvae have distinct immuno-histochemical properties from adult red fibers, the latter originate from the first (van Raamsdonk et al., 1982; Devoto et al., 1996; Buss and Drapeau, 2002). The red fibers are active at slow swimming frequencies and become de-activated at the highest frequencies of swimming, whereas the white fibers are active at higher frequencies but not slow frequencies (Buss and Drapeau, 2002). By 2 weeks, this muscle fiber composition has changed so that the adult red muscle fibers become fully differentiated and, in addition, intermediate muscle fibers have also appeared (van Raamsdonk et al., 1982; Devoto et al., 1996). These developmental changes in the musculature occur as the fish are becoming free-feeding and hence require a change in their behavioral repertoire (van Raamsdonk et al., 1982).

The change in the requirement of different behaviors across development is demonstrated by the developmental differences in the overall swim pattern of zebrafish. In larval zebrafish, the behavioral repertoire has been extensively studied and many “behavioral” categories have been identified using kinematic analyses (Olszewski et al., 2012; Mirat et al., 2013; Severi et al., 2014; Bianco and Engert, 2015; Temizer et al., 2015; Sternberg et al., 2016; Marques et al., 2018). When swimming, larval zebrafish use a so-called “burst and glide” manner, whereby a rapid burst of left-right alternating activity is followed by a gliding phase in which the body is kept straight (Saint-Amant and Drapeau, 1998; Budick and O’Malley, 2000; Buss and Drapeau, 2001). By contrast, adult zebrafish swim in a more continuous manner whereby the undulations of the body decrease in amplitude over the course of a swimming episode, rather than stopping intermittently (Müller et al., 2000; Gabriel et al., 2008). In adults, the next episode of swimming is often induced before the previous one has completely stopped, whereas larvae come to a complete halt before initiating movement anew (Müller et al., 2000).

## The Role of Brain-Descending Signals for Swimming

While spinal circuits generate the detailed rhythmic pattern underlying locomotor movements, their activity is initiated and controlled by descending signals from the mid- and hindbrain (Shik and Orlovsky, 1976; Dubuc et al., 2008; Jordan et al., 2008; Bouvier et al., 2015; Juvin et al., 2016; Capelli et al., 2017; Caggiano et al., 2018; Josset et al., 2018). Hindbrain reticulospinal neurons have been shown to be involved in turning, postural control and termination of swim activity (Deliagina et al., 2000; Grillner et al., 2008; Huang et al., 2013; Juvin et al., 2016); whereas the control of swimming in larval zebrafish has been associated with the most rostrally-located group of reticulospinal neurons, the nucleus of the medial longitudinal fasciculus (nMLF) in the midbrain. As targeted photoablation and optogenetic activation of subsets of nMLF neurons has shown, nMLF activity is crucial both for the control of swimming speed and swim steering by means of setting the tail angle (Severi et al., 2014; Thiele et al., 2014; Dal Maschio et al., 2017). Individual neurons of the nMLF project to different segment levels along the spinal cord where they connect to larval MNs (connections to INs have not been tested yet; Wang and McLean, 2014). Due to the low number of nMLF neurons, and to their characteristic topography and morphology, several of the neurons can be identified individually (Kimmel et al., 1982; Lee and Eaton, 1991; Gahtan and O’Malley, 2003; Thiele et al., 2014) and have been linked also to other behaviors such as prey capture (Gahtan et al., 2005). These studies have mainly focused on a few identified nMLF neurons at larval stages, but the roles of the other neurons are still unclear, as is the role of nMLF in adult zebrafish.

## Motoneuron Organization and Speed-Dependent Recruitment

Aside from ensuring transition between different behaviors, the spinal CPG also needs to accommodate for the smooth switch between different speeds of swimming. Utilization of the transcription factor code has uncovered a functional diversity of the MNs and the different classes of INs in the spinal cord of zebrafish (Kimura et al., 2006; McLean et al., 2008; Gabriel et al., 2011; Ausborn et al., 2012; Ampatzis et al., 2013; Björnfors and El Manira, 2016). Electrophysiology studies have shown that the two categories of MNs in zebrafish larva are differentially active during locomotion. The earlier born, primary MNs are active during fast swimming, escape and struggling behavior whereas the later born, secondary MNs are mainly active during slower swimming (see Figure 1 for the organization of the larval swim network; Myers, 1985; Myers et al., 1986; Westerfield et al., 1986; Liu and Westerfield, 1988; McLean et al., 2007). In the juvenile/adult zebrafish, this organization of the MNs has undergone some developmental changes in line with the changes in the muscle organization. At these stages, there are three distinguishable pools of secondary MNs which innervate slow, intermediate and fast muscles respectively, and are sequentially recruited as the swimming speed increases (see Figure 2 for an overview of the adult swim network; van Raamsdonk et al., 1982; Gabriel et al., 2011; Ampatzis et al., 2013). Interestingly, the primary MNs in the adult zebrafish, unlike in the larvae, no longer actively participate in normal swimming, but have become specialized as part of a separate circuit/module controlling the escape behavior (Ampatzis et al., 2013; Song et al., 2016).

The MNs are organized topographically, reflecting their order of recruitment as the speed of swimming increases. In the larvae, ventral MNs are recruited first at slow frequencies and more dorsal ones are added to the active pool as swim frequency increases (Figure 1). Thus, there is a linear relationship between recruitment frequency and dorso-ventral soma position in the spinal cord (Myers, 1985; Myers et al., 1986; Westerfield et al., 1986; Liu and Westerfield, 1988; McLean et al., 2007). This topographical organization remains into adulthood, with the three MNs pools occupying a specific location corresponding to a somatotopic map reflecting the type of muscle fibers the MNs innervate. In the adult, the motor column extends laterally and consequently MNs can be distinguished both by their dorso-ventral and latero-medial position (Figure 2). As swimming frequency increases, slow MNs located latero-ventrally are recruited first followed by intermediate ones located more medially, and finally fast MNs located more dorsally (Gabriel et al., 2011; Ampatzis et al., 2013). In addition to the segregation of soma position between the different types of MNs, their dendritic trees further distinguish them from one another. The adult fast MNs have more extensive dendritic arborizations which can extend into adjacent segments, whereas slow and intermediate MNs have dendritic arbors restricted to the same segment as that of the soma. Interestingly, the dendrites of the fast MNs have dorsally directed dendrites extending into neighboring segments, and ventrally directed ones which remain confined to the mother segment and appear to overlap with the dendritic arbors of the slow and intermediate MNs (Ampatzis et al., 2013). Studies in larval zebrafish have also shown that the dynamics of dendritic arborization in MNs correlate with their recruitment order. This study provides further evidence to a theory that neurons grow their dendritic arbors in accordance with the level of input required for their appropriate activity in the network (Kishore and Fetcho, 2013; see also Menelaou et al., 2014).

## V2a Interneurons as the Source of Excitation Underlying Locomotion

The excitatory drive to MNs emanates from ipsilateral excitatory INs underlying the on-cycle depolarization, with commissural INs underlying mid-cycle inhibition to ensure the alternating pattern between the two sides of the spinal cord (Harris-Warrick and Cohen, 1985; Burke et al., 2001; Grillner, 2003; Brownstone and Wilson, 2008; Goulding, 2009; Kiehn, 2016; Ziskind-Conhaim and Hochman, 2017). Excitatory INs represent the core components for the generation of the locomotor rhythm. A first step to understanding the organization of the locomotor CPG is the identification of the excitatory INs generating the locomotor rhythm. The focus has been on the V2a INs with properties reminiscent of the excitatory INs identified in lamprey (Buchanan and Grillner, 1987; Buchanan et al., 1989; Buchanan, 1993, 1996) and *Xenopus* (Dale and Roberts, 1985; Soffe et al., 2009; Li and Moult, 2012). Ablation- and optogenetic experiments in larval zebrafish have shown that V2a INs are both necessary and sufficient for the generation of locomotion. Acute two-photon ablation of V2a INs over ten segments in the mid-body region prevented the generation of the locomotor rhythm. (Eklöf-Ljunggren et al., 2012). In addition, optogenetic activation of V2a INs in the spinal cord of larval zebrafish was sufficient to induce coordinated locomotor rhythm (Eklöf-Ljunggren et al., 2014). Furthermore, selective ablation of dorsal V2a INs only prevented the generation of swimming activity at higher speeds, while ablation of the ventral V2a INs abolished the generation of swimming at all speeds. These early findings were corroborated using genetic silencing of synaptic transmission from V2a INs both on fast and slow swimming (Sternberg et al., 2016). These results suggest that the different sets of V2a INs are necessary for the generation of swimming at different speeds and that ventral/slow V2a INs are required for the initiation of locomotion, since without them no rhythmic activity could be initiated. Taken together, these studies suggest that the V2a INs are the main source of excitatory drive in the spinal cord. In line with these findings, a cell type called ipsilateral caudal cells (IC) in zebrafish embryos have been shown to drive an immature type of motor behavior called coiling. A bit further into development, the IC cells display sustained bursting which enables locomotor oscillations of high frequency as the embryo develops into a swimming larva (Tong and McDearmid, 2012).

## Functional Diversity of V2a Interneurons and Modular Circuit Organization

The V2a INs have been well characterized in terms of their activity pattern and morphology, both in the larval and the adult zebrafish. These INs are differentially active depending on their birth order. In the larva, earlier born neurons have high input resistances and are active during faster swimming and escapes, and later born neurons have low input resistances and are active at slower swimming frequencies (Kimura et al., 2006; McLean et al., 2008; McLean and Fetcho, 2009). In addition, these V2a INs follow a specific recruitment pattern. The later born ventral neurons, which are active at low frequencies, become inactive at higher frequencies while earlier born, dorsal, neurons become active at higher frequencies (Figure 1; McLean et al., 2007, 2008). This recruitment pattern suggests that larval zebrafish INs are active at specific frequency spans of swimming and that they are arranged topographically.

In adult zebrafish, the V2a INs still display diversity in their activity pattern since they are activated in a speed-dependent manner; however, this segregation now follows that of the MNs, such that they are divided into slow, intermediate and fast depending on the lowest frequency at which they become active. Moreover, adult zebrafish V2a INs are not inhibited (de-recruited) at specific frequencies but rather remain active once above recruitment threshold (Ausborn et al., 2012). Interestingly, adult V2a IN somata appear to have lost their topographic arrangement (Figure 2). Instead their recruitment depends on a balance between the input they receive and their intrinsic properties (Ausborn et al., 2012).

The V2a INs have been described to be mainly descending, with a subset also displaying ascending projections (Kimura et al., 2006; Eklöf-Ljunggren et al., 2014; Menelaou et al., 2014). In larval zebrafish, anatomical studies have shown differences in projection patterns and synapse location in V2a INs involved in different swim speeds. Such anatomical discrepancies point at potential differences in connectivity between MNs and V2a INs recruited at different speeds (Menelaou et al., 2014). It has also been suggested that fast V2a INs are connected to the fast primary MNs in a specific topographic manner with one ventral and one dorsal stream. This allows a selective control of the activity of primary MNs innervating the dorsal and ventral parts of the myotome (Bagnall and McLean, 2014). These connections appear to be restricted to electrical but not to chemical synapses. A recent anatomical study using serial-block electron microscopy reconstructed and mapped all the connections between the descending V2a INs and MNs within a single spinal segment in a larval zebrafish (Svara et al., 2018). The authors found that the dorsal-most descending V2a INs were selectively connected to primary MNs, whilst the more ventral V2a INs had less specificity in their connections with MN subtypes. Interestingly, they also identified that the smallest, most ventral MNs were present in the larval zebrafish spinal cord, but they lacked dendrites, and received little to no synaptic input from any V2a INs. This suggests that in larval zebrafish, slow MNs are yet to be integrated into the swim network, and presumably become wired into the network as development progresses (Svara et al., 2018).

Paired recordings between MNs and V2a INs in the larval zebrafish have shown that they have monosynaptic connections, which are both chemical and electrical. Furthermore, synaptic strength appeared to be stronger between neurons involved in faster or stronger movements than in those involved in weaker movements (Kimura et al., 2006). This appears to hold true into adulthood where fast MNs and V2a INs have stronger excitatory drive and electrical coupling coefficients than their slow or intermediate counterparts (Ampatzis et al., 2014; Song et al., 2016).

In the adult zebrafish, the V2a IN class underlying the swimming rhythm is heterogeneous with three sub-classes that are driving locomotion at different speeds and are connected preferentially to the different MN pools to form three separate speed circuit modules. At slow speeds, up to 3–4 Hz, only the slow V2a INs are engaged. These INs are preferentially connected to slow MNs forming a slow speed circuit sufficient to sustain slow swimming. Once the swimming speed exceeds 4 Hz, intermediate V2a INs become activated to increase the speed up to ~8 Hz. These intermediate V2a INs are also preferentially connected to intermediate MNs and form a separate intermediate speed module. Finally, during fast swimming the fast V2a INs are deployed and drive the activity of fast MNs, forming the fast speed circuit module (Ampatzis et al., 2014). Hence, this functional diversity of the V2a INs and MNs endows the adult zebrafish locomotor CPG with a modular organization encompassing three speed modules activated sequentially to control the speed of swimming.

Heterogeneity of V2a IN firing pattern, and their recruitment during locomotion, has also been reported in the mouse spinal cord (Dougherty and Kiehn, 2010; Zhong et al., 2010, 2011). In addition, there is anatomical evidence that V2a INs make synaptic contacts with MNs in the mouse spinal cord (Ruder et al., 2016; Capelli et al., 2017). Therefore, it is likely that the modular circuit organization revealed in zebrafish could also hold for the organization of the locomotor CPG in mammals.

Remarkably, the drive from V2a INs to MNs is directly influenced by MNs’ activity that is transmitted retrogradely via gap junction coupling. In the adult zebrafish, V2a INs make mixed electrical and chemical synapses onto MNs. This enables MNs to retrogradely influence the activity of the upstream V2a INs. Such retrograde influence shows that the zebrafish MNs are not passive recipients of commands elaborated by premotor INs, but an integrated component of the network generating locomotion (Song et al., 2016). The involvement of MNs in the generation of the locomotor rhythm has been revealed in mammals and lamprey, whereby retrograde messengers such as glutamate and endocannabinoids (eCBs) are released by MNs to influence the activity of the INs driving them (Kettunen et al., 2005; Kyriakatos and El Manira, 2007; Falgairolle et al., 2017; see also Bhumbra and Beato, 2018).

## V0 Interneuron Diversity and Function

An important commissural IN class in the spinal cord is the V0 population, which comprises the excitatory glutamatergic V0v and the inhibitory (glycinergic and GABAergic) V0d INs (Moran-Rivard et al., 2001; Pierani et al., 2001; Lanuza et al., 2004; Satou et al., 2012; Talpalar et al., 2013; Björnfors and El Manira, 2016). These two V0 sub-classes are produced from different progenitors (Moran-Rivard et al., 2001; Pierani et al., 2001; Lanuza et al., 2004; Satou et al., 2012). In the larval zebrafish, the inhibitory V0d INs (CoBL) have a relatively stereotyped morphology, with both glycinergic and GABAergic subtypes displaying bifurcating axons. The larval excitatory V0v INs, on the other hand, could be categorized into three subtypes based on their axonal trajectories; V0v ascending (V0v-A), V0v bifurcating (V0v-B) and V0v descending (V0v-D). The V0v-A are produced from distinct progenitors, while V0v-B and V0v-D INs are produced from common progenitors. Furthermore, the different V0v subtypes are differentiated in a precise temporal order with the V0v-A appearing first, followed by V0v-B and finally V0v-D (Satou et al., 2012). Electrophysiological analysis of the activity of one type of V0v-D INs (MCoDs) has shown that these neurons are active only during slow swimming and become inhibited when the swimming switches to higher speeds (Figure 1; McLean et al., 2007, 2008). However, the function of the V0v-A and V0v-D has not been examined in larval zebrafish.

In the adult zebrafish, a detailed analysis of the pattern of recruitment of V0v INs combined with morphology and axonal trajectories has revealed a greater diversity of V0v INs (Björnfors and El Manira, 2016). Like MNs and V2a INs, the V0v INs in adult zebrafish are also subdivided into three speed-dependent subtypes (slow, intermediate and fast; Figure 2). In addition, a fourth subtype of V0v IN was observed which did not receive rhythmic input during locomotion. The majority of the V0v INs examined in the adult belong to the fast subtype. Furthermore, while all the three morphological subtypes of V0v described in larval zebrafish were also found in the adult, they were not distinctive of any of the three speed-dependent subtypes, and could be ascending, descending, bifurcating or local (Björnfors and El Manira, 2016). Thus, the combined analyses in larval and adult zebrafish have revealed a large developmental, morphological and functional diversity of the V0v INs (Satou et al., 2012; Björnfors and El Manira, 2016).

## Escape Circuit and Behavioral Choice

**Figure 3:**
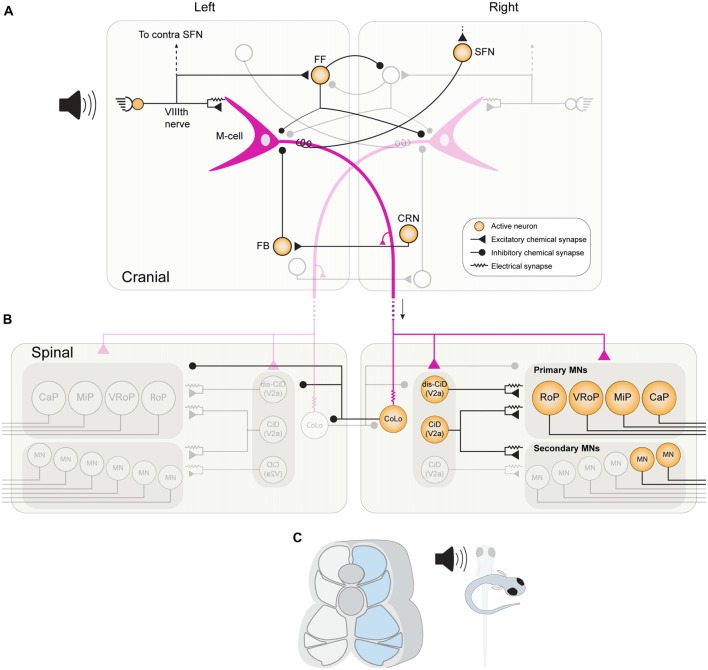
The brainstem and spinal cord network in larval zebrafish for mediating a fast-onset C start response. **(A)** An acoustic stimulus from the left stimulates afferent hair cells, which provide direct excitation to the left M-cell (purple) via mixed chemical and electrical synaptic boutons. Afferent hair cells also provide convergent excitation to the ipsilateral M-cell via activation of spiral fiber neurons (SFNs). The afferent hair cells also stimulate a population of feedforward (FF) inhibitory INs, which mostly inhibit the contralateral M-cell and contralateral FF inhibitory neurons. As the M-cell spike propagates down its axon it stimulates cranial relay neurons (CRNs) via axon collaterals, which activate feedback (FB) inhibitory INs that prevent the M-cell from generating consecutive spikes. **(B)** When the M-cell axon reaches the spinal cord, it provides direct chemical excitation of primary MNs on the right side, as well as to excitatory CiD INs. CiD INs provide further excitation via mixed chemical and electrical synapses to primary and fast secondary MNs. Finally, the M-cell axon also rapidly excites inhibitory Commissural local INs (CoLos) via electrical synapses, which in turn inhibit various MNs and INs on the contralateral side. **(C)** The activation of this escape network results in strong contraction of fast musculature on the right side of the body, resulting in fish curving into the characteristic C-shape.

Zebrafish mainly navigate their natural environment using the spinal swim network outlined above. However, certain circumstances, such as a threat from a predator, demand a more rapid behavioral response which cannot be accommodated by the swim network alone. When inbound sensory information indicates a threat, such as the sound, sight or movement of a predator, teleost fish perform an extremely fast escape maneuver known as a “startle response.” This involves the relay of sensory information to populations of reticulospinal neurons (RSNs), including the command-like Mauthner neuron, which in turn excite a dedicated network of neurons in the spinal cord that implements a behavioral response directing the fish away from a threat (Figure 3).

Teleost fish most commonly perform a startle response known as the “C-start” maneuver, which involves the rapid contraction of axial muscles on the side contralateral to the stimulus (Eaton et al., 1977). This is followed by counter-bending in the opposite direction that propels the animal forwards, and is often seamlessly followed by a phase of fast swimming. The reliability and rapidity of the C-start is accommodated by a dedicated escape circuit in the brainstem and spinal cord which has been studied intensively across numerous fish species, especially in goldfish (for reviews, see Zottoli and Faber, 2000; Eaton et al., 2001). Furthermore, studies across fish species are revealing that there is considerable flexibility in the escape response depending on the environmental context (Domenici, 2010; Machnik et al., 2018a,b). Recent studies using the zebrafish have continued to illuminate new insights into the neural mechanisms underlying the C-start response and its inherent flexibility. It has also been shown that larval zebrafish display a second form of startle, the so-called “S-start” maneuver, which is implemented by local inhibitory networks in the spinal cord (Liu et al., 2012). Considerable progress has been made using different developmental stages of zebrafish to understand how the directionality, intensity and speed of the startle response is influenced by the direction (head/tail; left/right) and modality (tactile/auditory/visual) of the sensory stimulus, as well as the complex networks of neurons involved.

### Reticulospinal Neurons: M-Cell and Non-M-Cell Mediated Escape

The C-start response in teleost fishes is usually triggered by a single action potential in one of a bilateral pair of large brainstem RSNs known as Mauthner cells (M-cells: Mauthner, 1859; Zottoli, 1977). M-cells act as sensory integrators that receive inputs from the vestibular and auditory branches of the VIIIth cranial nerve, as well as from the lateral line and optic tectum (Eaton and Farley, 1975; Faber et al., 1991). The axon of each M-cell descends to the contralateral side of the fish, permeates the entire length of the spinal cord, and provides excitatory input to primary MNs and various spinal INs that implement the escape behavior (Figure 3; Eaton and Farley, 1973; Fetcho and Faber, 1988).

Several IN populations impinge on the M-cells (Figure 3), and recent work in larval zebrafish has begun to unravel the function of these populations in modulating M-cell excitability (Koyama et al., 2011). One such population, known as “spiral fiber neurons” (SFNs), are activated by startle-inducing stimuli and provide feedforward (FF) excitatory input to the contralateral M-cell via both electrical and glutamatergic chemical synapses (Koyama et al., 2011). Ablation of SFNs reduces the occurrence of short-latency C-start responses, whilst their optogenetic activation has the opposite effect, demonstrating that they provide a convergent pathway for M-cell activation (Lacoste et al., 2015). M-cells also receive FF inhibition from a population of glycinergic neurons, which inhibit both the ipsilateral and contralateral M-cells, as well as reciprocally inhibiting each other. A recent study in zebrafish identified two key roles for this inhibition (Koyama et al., 2016). First, FF inhibition decreases the overall sensitivity of both M-cells, thus preventing escape responses being triggered by weak, non-salient stimuli. Second, FF inhibition is critical for generating the appropriate left-right directionality of a C-start. Although the inhibitory populations target both M-cells, the inhibition to the contralateral M-cell is stronger. Thus, when sensory information reaches one side of the animal, not only do numerous excitatory pathways converge onto the ipsilateral M-cell, but FF inhibition shuts off the contralateral M-cell, whilst simultaneously disinhibiting the ipsilateral M-cell. The M-cells also receive feedback inhibition from a separate population after they have fired an action potential. Cranial relay neurons (CRNs) receive excitation from the M-cell axon and in turn excite a population of feedback glycinergic inhibitory neurons. These provide inhibition to the axon cap of the M-cell and prevent the induction of subsequent M-cell spiking after a C-start, which could potentially disrupt the ongoing startle response (Koyama et al., 2011).

The M-cell is critical, and always activated, during a tail-evoked C-start response in larval zebrafish (O’Malley et al., 1996; Liu and Fetcho, 1999). However, for head-directed stimuli, C-starts can also be elicited in the apparent absence of M-cell activation (O’Malley et al., 1996), and are observed in larval zebrafish in which the M-cell has been ablated (Liu and Fetcho, 1999; Kohashi and Oda, 2008). These so-called non-M-cell C-starts occur with a slightly delayed onset, but are otherwise identical to an M-cell C-start. The neurons responsible for this non-M-cell response are bi-laterally paired segmental homologs to the M-cell, MiD2cm and MiD3cm, located in rhombomere 5 and 6, respectively (collectively known as the “M-series”; Lee and Eaton, 1991). The M-cell homologs supplement the M-cell in mediating head-evoked startle behavior, and recent studies using *in vivo* calcium imaging in head-embedded larval zebrafish have revealed stimulus- and development-dependent conditions in which different members of the M-series become activated (Kohashi and Oda, 2008; Kohashi et al., 2012). In larval zebrafish, fast-onset C-start responses, mediated solely by the M-cell, were found to be elicited by acoustic-vestibular stimuli. On the other hand, head-tactile stimuli elicited slow-onset C-start responses, which involved the activation of MiD3cm, but *not* the M-cell (non-M-escape). Interestingly, newly hatched zebrafish (<75 hpf) generate C-start responses only to head-tactile stimuli, which involves the activation of the full M-series, suggesting that as development progresses, the reticulospinal network becomes responsive to new stimuli and the M-series more refined for specific sensory inputs (Kohashi et al., 2012).

### Spinal Networks for Escape

In both larval and adult zebrafish, the axon of the M-cell enters the spinal cord and makes monosynaptic connections with the primary MNs that innervate fast white muscle, which provide the powerful driving force that initiates the C-bend (Myers, 1985; Westerfield et al., 1986; Fetcho, 1991). In larval zebrafish, primary MNs also participate in normal swimming, whereas in the adult they have become dedicated to escape (Ampatzis et al., 2013; Song et al., 2016). The M-cell axon not only contacts the primary MNs, but also various spinal INs, some of which overlap with those involved in normal swimming. For example, in zebrafish larvae, dorsally-located glutamatergic CiD INs receive input from the M-cell axon and provide both chemical and electrical input to fast-type secondary MNs, as well as primary MNs, thus supplementing the excitatory drive for muscle contraction (Kimura et al., 2006).

A number of inhibitory INs are also activated by the M-cell, including glycinergic CoBL INs and CoSA sensory INs, both of which are active during normal larval swimming as well (Liao and Fetcho, 2008). However, one spinal inhibitory population is known to be dedicated to the escape response. Commissural local INs (CoLos) are recruited *only* during the escape behavior, and never during normal swimming or struggling (Liao and Fetcho, 2008; Satou et al., 2009). CoLos receive rapid excitation from the M-cell axon via electrical synapses and inhibit the contralateral MNs via glycinergic chemical inputs (Figure 3). CoLos also simultaneously inhibit contralateral CiD and CoLo INs via glycinergic chemical synapses, thus act not only by inhibiting the driving force for contralateral muscle, but also disinhibiting the neuronal populations on the ipsilateral side. Occasionally, both M-cells can fire simultaneously in response to a head-evoked stimulus and the downstream CoLos function to ensure that only muscles on the contralateral side to the earliest-firing M-cell are able to contract (Satou et al., 2009).

### A Local Spinal Circuit for the S-Start Response

The C-start is a rapid and efficient behavioral sequence that orients the fish in the opposite direction to a threat, and is therefore perfectly suited to evading rostrally-located stimuli. However, a second startle response, the S-start, is frequently observed in zebrafish larvae in response to tail-evoked stimuli (Liu et al., 2012). Both the S-start and C-start are comparable in speed and timing and both involve M-cell activation. However, whilst C-starts often involve the activation of other RSNs, only the M-cell appears to be involved in S-starts (O’Malley et al., 1996; Liu and Fetcho, 1999). There are therefore distinctions between neuron recruitment even at the level of the brainstem, but local circuits in the spinal cord are also key in implementing either the C-start or S-start.

Recent research in larval zebrafish has identified how local inhibitory circuits in the spinal cord act to bias the network towards performing either a C-start or an S-start maneuver in response to a tail-directed stimulus (Liu and Hale, 2017). Unusually, the S-start is mediated by simultaneous activation of both M-cells, which is important as the S-start requires near simultaneous contraction of rostral and caudal muscles on contralateral sides of the fish. When an S-start is observed, however, primary MNs in the caudal spinal cord were shown to selectively receive inhibition that preceded the firing of the M-cells. It turns out that immediately prior to M-cell activation, Rohon-Beard (RB) sensory neurons that detect a tail-stimulus excite caudally-located inhibitory CoLos (described above). Once the M-cells do then fire, the first M-cell to spike activates the ipsilateral, rostral musculature, and inhibits the contralateral rostral musculature (as in the normal C-start response). However, once the signal reaches the caudal spinal cord, the side that is activated depends upon the extent to which CoLos have been disengaged by RB neurons. If the CoLos are most active on the stimulus side, a normal C-start occurs, but if RB neurons have actively engaged the contralateral CoLos, the muscle activation switches sides and an S-start is generated. A local, inhibitory circuit in the caudal spinal cord is thus superimposed onto the C-start network, whose activation dictates whether a descending command for escape is transformed into a C-start, or an S-start maneuver. Interestingly, S-start responses are not observed in adult zebrafish (Liu and Hale, 2017). One possible explanation for this is that the loss of RB neurons during zebrafish development (Kanungo et al., 2009) removes the local inhibitory circuit that is crucial for generating the S-start response.

## Modulation of the Swimming and Escape Circuits

While the previously described spinal cord modules form hard-wired circuits that organize swimming and escape at different speeds, an additional way to obtain operational flexibility of swim circuits is through their modulation. Modulation allows a dynamic regulation of the networks, reconfiguring them to produce different outputs which can be achieved in multiple ways, like modulation by sensory inputs or neuromodulators. Neuromodulators have been described to shape the output of locomotor circuits from invertebrates to mammals (Harris-Warrick, 2011; Marder, 2012; Sillar et al., 2014; Nusbaum et al., 2017).

**Figure 4:**
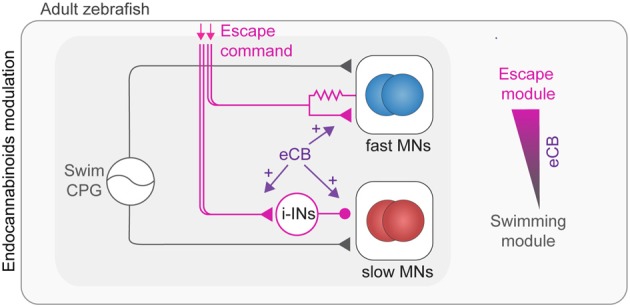
Modulation of adult swimming and escape circuits by endocannabinoids (eCBs). Swimming behavior is generated by speed dependent excitation of MNs by the spinal swim CPG network (gray connections). Activation of the escape network (purple connections) leads to temporary interruption of swim behavior. The escape command leads to a switch from slow to fast MN pools which is mediated by direct excitation of fast (blue) and indirect inhibition of slow MNs (red). The selection of escape over swimming behavior is positively modulated by eCB through enhancement of synaptic transmission.

ECBs represent an important neuromodulatory system which plays a crucial role in behavioral selection between escape and swimming as well as in the modulation of the swimming speed (Figure 4; Cachope et al., 2007; Song et al., 2015). To allow for efficient behavioral selection, the CNS is endowed with circuit mechanisms ensuring a behavioral hierarchy. For example, the perception of danger elicits fast escape behavior that overrides other competing motor behaviors such as feeding or slow locomotion (McCleery, 1983; Winlow et al., 1992; Kristan, 2008; Liden et al., 2010; Yilmaz and Meister, 2013; Pirger et al., 2014). In zebrafish, the induction of escape by the Mauthner system transiently interrupts ongoing swimming, which resumes upon termination of escape (Fetcho, 1991; Song et al., 2015). This selection of escape over swimming is mediated by switching between fast and slow MN pools. Fast MNs underlying escape become rapidly recruited via direct excitation from the Mauthner system, whereas the slow MNs underlying swimming become derecruited via indirect inhibition that operates in a clutch-like manner to decouple these MNs from the swimming CPG (Figure 4). This hardwired circuit for behavioral choice is under a strong eCB modulation that sets the threshold for initiation of escape and determines the duration of the interruption of swimming (Song et al., 2015). The eCB modulation shifts the balance between excitation and inhibition (strengthening excitation) to ensure execution of the proper escape behavior and block out any interference of swimming. The eCB involved is released from MNs and acts retrogradely to modulate synaptic transmission (Figure 4) as well as setting the baseline swimming frequency as previously shown in lamprey spinal cord (Kettunen et al., 2005; Kyriakatos and El Manira, 2007; Song et al., 2012). While the generally established effect of eCB is depression of synaptic transmission (Kano et al., 2009; Castillo et al., 2012), the effect on the switch between the swim modules is realized by the potentiation of synaptic transmission.

The circuit and eCB mechanisms mediating the primacy of escape over swimming also underlies the behavioral selection that is related to the social status of animals where dominant zebrafish mostly perform slow swimming movements while subordinate fish display primarily fast escape behavior (Miller et al., 2017).

Dopamine has been shown to influence overall swim levels in zebrafish, although its complete action remains to be fully understood. In vertebrates, including zebrafish, the sole source of dopamine in the spinal cord are diencephalospinal neurons (DDNs; McLean and Fetcho, 2004; Filippi et al., 2010; Tay et al., 2011). Ablating DDNs suppressed swimming, as shown by shortened travel distances (Lambert et al., 2012; Jay et al., 2015) due to a reduction in the overall time that the zebrafish spent swimming (Jay et al., 2015). While the swim speed remained the same (Jay et al., 2015), it is still a matter of debate whether other parameters of the swim pattern, such as episode duration are affected by dopamine (Lambert et al., 2012; Jay et al., 2015; Lambert, 2016). DDT neurons are thought to act via spinal D1 and D4 receptors (Lambert et al., 2012). Generally, dopamine has been shown to have a differential effect on locomotion depending on the respective target receptors (D1–D5) in brain and spinal cord and depending on the developmental stage (Thirumalai and Cline, 2008; Lambert et al., 2012).

Finally, another aminergic neuromodulator, serotonin, has been shown to increase the overall motor output in zebrafish (Brustein et al., 2003). The serotonergic system, like other aminergic ones, is in place shortly after hatching in zebrafish (Brustein et al., 2003; McLean and Fetcho, 2004). However, the serotonergic modulation of the locomotor activity and the underlying mechanisms undergo developmental changes as serotonin has different effects at early and late developmental stages. At early stages, serotonin increased the overall swimming activity by reducing the pauses between swimming episodes without affecting the burst frequency or the duration of the swimming episodes (Brustein et al., 2003). The modulation of the locomotor activity in zebrafish larvae is mediated by modulation of chloride homeostasis, which regulates the quiescent period between swimming episodes (Brustein and Drapeau, 2005). In juvenile and adult stages, serotonin decreased the frequency of swimming (Gabriel et al., 2009). The decrease in the swimming frequency is mediated by potentiation of mid-cycle inhibition that results in a delayed onset of the subsequent swimming burst (Gabriel et al., 2009).

Overall, neuromodulators seem to manipulate swimming in multiple ways and some of them undergo developmental changes in their way to affect the locomotor network and their mechanisms used. Some neuromodulators do so by specifically acting on individual circuit modules, while for many others, their mechanism of action on swimming remains to be determined.

## Sensory Modulation to Spinal CPG Modules

Once initiated, the swimming activity generated by the CPG networks can be sustained in the absence of any sensory afferent inputs. Deprived of any peripheral input, the neural networks in the isolated spinal cord can produce locomotion in many species (Delcomyn, 1980; Grillner et al., 1981). However, when blocking the neuromuscular junctions in the larval zebrafish preparation, the swimming frequency is lower than the spontaneous swimming frequency (Knafo et al., 2017), indicating that proprioception has a modulatory role in shaping locomotor activity and speed control. Similarly, the sensory-motor integration needed for limb movement, which is well known in tetrapods, has its correspondent in the fish pectoral fin network (in sunfish, *Lepomis macrochirus*). The sensory input is not necessary for swimming itself, but it is involved in movement speed and posture of the fins (Williams and Hale, 2015). Since the preparation used for larval zebrafish electrophysiological experiments includes intact muscles as well as sensory system, and can elicit a range of different behaviors, it is an ideal model to study the interactions between sensory and motor circuits. We will here specifically point out the studies which start to shed light on the fine connectivity of the sensory-motor spinal circuits and the effects on the motor output in larval zebrafish.

The main sensory components contributing to the larval zebrafish spinal circuits are the spinal mechanosensory neurons, or RB and the CSF-CNs neurons. The first provide mechanosensory feedback from the periphery, proprioception and nociception, the latter are intraspinal sensory neurons responding to pH changes and lateral bending (as reviewed in Böhm and Wyart, 2016). Although another sensory component giving feedback to the motor system from the periphery is the lateral line (Liao, 2010; Oteiza et al., 2017), it will not be discussed in this review as it relays information to the brain and not directly to the spinal CPGs (Liao, 2010).

**Figure 5:**
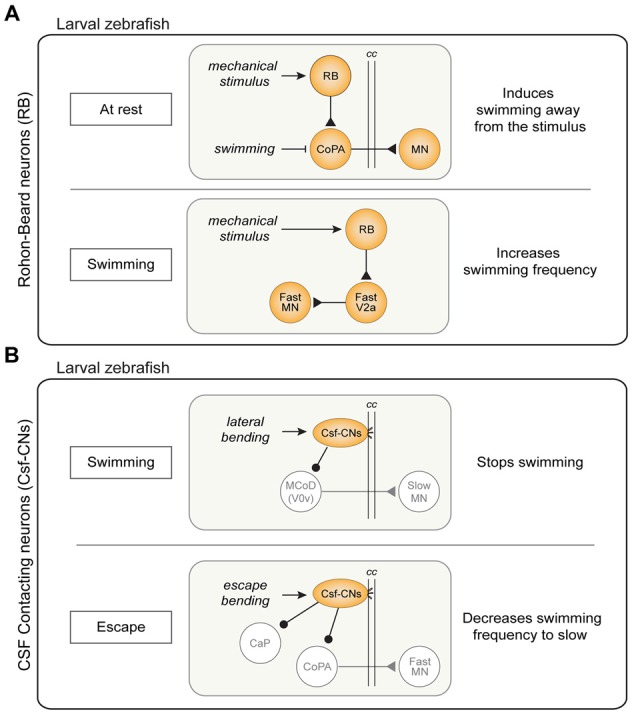
Sensory modulation of swimming and escape circuits in larval zebrafish. **(A)** Pathways of Rohon-Beard (RB) neurons activity. Top: disynaptic cutaneous skin reflex. When the fish is at rest, an activation of RB sensory neurons results in swimming away from the stimulus through the excitation of commissural primary ascending (CoPA) INs. This mechanism is inhibited by during ongoing swimming. Bottom: during swimming, activation of RB sensory neurons act on the fast system increasing swimming frequency through the excitation of dorsal V2a INs. **(B)** Cerebrospinal fluid-contacting neurons (CSF-CNs) activity pathways. Top: lateral bending of the spinal cord during swimming activates CSF-CNs which respond inhibiting the MCoD (V0v) INs therefore stopping locomotion. Bottom: CSF-CNs are activated during escape behavior and act on the fast system inhibiting caudal primary MNs (CaP) and the CoPA INs, decreasing the swimming frequency. cc: central canal.

In zebrafish, RB neurons are a larval feature, replaced in juveniles by the dorsal root ganglion (DRG) neurons (Kanungo et al., 2009). Based on our current knowledge, RB neurons selectively modulate fast swimming and escape behaviors with two distinct pathways involving a population of commissural INs (CoPA) or V2a INs, respectively. The first pathway is the well-known disynaptic cutaneous skin reflex, a simple and conserved mechanism found in *Xenopus*, zebrafish larvae and lamprey (Buchanan and Cohen, 1982; Clarke et al., 1984; Christenson et al., 1988; Li et al., 2003; Pietri et al., 2009; Easley-Neal et al., 2013). When the skin is stimulated at rest, RB neurons activate CoPA neurons, which in turn drive contralateral MNs to produce swimming away from the stimulus (Figure 5A; Buchanan and Cohen, 1982; Christenson et al., 1988; Li et al., 2003). During normal swimming this mechanism is inhibited at CoPA neurons level to avoid activation of the cutaneous reflex by movement (Sillar and Roberts, 1988; Knogler and Drapeau, 2014). The second pathway consists in a presumably monosynaptic connection between RB neurons and the subset of V2a INs that drives fast swimming. The mechanosensory feedback signaled by RB neurons enhances specifically the fast swim regime, thereby increasing the locomotor frequency, and promoting a faster escape (Figure 5A). Coherently with these results, silencing of RB neurons in freely swimming larvae reduces tail-beat frequency and speed of the locomotor output (Knafo et al., 2017). The lack of activation of mechanosensory neurons could explain the lower frequency span obtained in isolated spinal cord preparation compared to the spontaneous swimming frequencies in the intact preparation.

The second sensory input which modulates spinal locomotor networks comes from CSF-CNs. These ventral intraspinal GABAergic sensory neurons, described for the first time almost 100 years ago are present in more than 200 species of vertebrates (Kolmer, 1921; Agduhr, 1922; Dale et al., 1987; Djenoune et al., 2014). They have a characteristic shape with cilia protruding in the central canal (cc) that sense lateral bending as well as acidification of the CSF through a mechanosensory channel (in mouse, zebrafish and lamprey; Djenoune et al., 2014; Böhm et al., 2016; Jalalvand et al., 2016a,b). Zebrafish larvae with a mutated form of this channel show a reduction of tail beat frequency during the escape (Böhm et al., 2016). In lamprey, lower pH leads to a reduction in locomotor burst frequency, due to the CSF-CNs decreasing the activity in the locomotor network (Jalalvand et al., 2016b). In larval zebrafish, by contrast, optogenetic activation of the intraspinal sensory neurons was initially thought to produce swimming (Wyart et al., 2009). This view was subsequently revised as it turned out that the GABAergic CSF-CNs actually inhibit ongoing swimming and the previous reported implication in inducing swimming was in fact a rebound following inhibition (Fidelin et al., 2015). Indeed, when the background excitation was increased optogenetic stimulation of CSF-CNs induced a rebound slow swimming after a delay of 450 ms from the end of their stimulation (Wyart et al., 2009; Fidelin et al., 2015; Sternberg et al., 2016).

The CSF-CNs act directly on the premotor network both on slow and fast excitatory commissural INs (MCoD and CoPA respectively) as well as on a specific type of fast primary MNs (CaP). The inhibition of the MCoD INs (a subtype of V0v INs) is thought to mediate the inhibitory effect of CSF-CNs on slow swimming (Figure 5B; Fidelin et al., 2015). On the other hand, CoPA INs and CaP MNs are important for escape and their inhibition by CSF-CNs is thought to help maintain the postural control during fast swimming and escape (Hubbard et al., 2016; Sternberg et al., 2016). In natural conditions, the CSF-CNs forms a mechanosensory system detecting longitudinal spinal cord bending and providing inhibitory tone to CaP MNs (Figure 5B). It has been proposed that the inhibitory tone also serves as postural control; indeed, silencing CSF-CNs leads to a postural control defect during escape (Hubbard et al., 2016). Summarizing, after a C-bend accompanied by a strong CaP activation, the CSF-CNs strongly inhibit CaP MNs, switching the behavioral outcome to slow swimming frequencies. The synaptic depression mechanism is presumably functioning to avoid that the inhibition arises during swimming (Hubbard et al., 2016).

In conclusion, the CSF-CNs represent a novel sensory system that is activated by both movement and change in the acidity and hence help control in an activity-dependent manner the operation of the locomotor networks to adapt to its internal state.

## Conclusion and Perspectives

The goal of motor control neuroscience is to understand how behavior arises from activity patterns in the neural circuits of the brain and spinal cord. Decoding the organization and function of locomotor circuits has been rapidly accelerated by the (often combinatorial) use of genetic, cellular, circuit and behavioral approaches across a range of species, and many principles have emerged that inform our understanding of species across the vertebrate lineage. Zebrafish offer numerous strengths as a model organism, including the availability of mutant zebrafish with increasingly refined expression of fluorescent markers and optogenetic proteins; a transparency that allows for optical approaches during ongoing behavior in the intact animal; and the opportunity to apply these approaches in both larval and adult preparations. As such, zebrafish research has been especially fruitful in revealing new insights into the locomotor circuit, and in highlighting the similarities, minor modifications and transformations of the locomotor circuitry through development.

In this review article, we have highlighted several aspects of locomotion, where recent investigations in zebrafish across developmental stages have provided new insights into how the brain and spinal circuits are organized, and modulated, to endow zebrafish with a large behavioral repertoire. In particular, the electrophysiological, anatomical and functional characterization of transcriptionally-defined V2a INs has revealed them to be the source of excitatory drive for locomotion in larval and adult zebrafish. Furthermore, parallel work in larvae and adult has revealed specific rules of connectivity between V2a INs and MN pools, which enables flexibility in speed control according to the behavioral needs at a given period of development. This work has also elaborated our understanding of how premotor excitatory neurons interact with MNs via gap junctions, highlighting the MNs as a critical component of the rhythm-generation machinery.

There are, however, many outstanding issues that need to be resolved. The function and connectivity of other genetically-defined spinal IN groups are only partially understood. Another unresolved issue is linking the diversity of spinal neurons, and their organization into task-specific circuits, to their role in providing the versatility of motor behavior. As the techniques for classifying spinal neuron types is becoming increasingly sophisticated, studies in zebrafish will continue to contribute to solving some of these issues in the motor control field.

## Author Contributions

EMB, ERB, IP and LP wrote the first draft of the review and prepared the figures. AE revised the review and figures together with all authors.

## Conflict of Interest Statement

The authors declare that the research was conducted in the absence of any commercial or financial relationships that could be construed as a potential conflict of interest.
